# Integrating conservation agriculture with intensive crop diversification in the maize-based organic system: Impact on sustaining food and nutritional security

**DOI:** 10.3389/fnut.2023.1137247

**Published:** 2023-03-14

**Authors:** Meraj Alam Ansari, N. Ravisankar, Majhrool Hak Ansari, Subhash Babu, Jayanta Layek, A. S. Panwar

**Affiliations:** ^1^ICAR Research Complex for NEH Region, Manipur Centre, Imphal, India; ^2^Coordination Unit, ICAR-Indian Institute of Farming System Research, Meerut, India; ^3^Faculty of Agricultural Sciences, Aligarh Muslim University, Aligarh, India; ^4^Division of Agronomy, ICAR-Indian Agricultural Research Institute, New Delhi, India; ^5^ICAR Research Complex for NEH Region, Meghalaya, India

**Keywords:** conservation agriculture, crop intensification, dietary energy returns, food security, production economics, nutritional security

## Abstract

**Introduction:**

Developing an intensive sustainable model and feeding a rising population are worldwide challenges. The task is much more daunting in the North Eastern Himalayas, where, low productive maize (*Zea mays*)- fallow is the main production system in the upland. To increase farm productivity, nutritional security, and energy dietary returns while maintaining environmental sustainability and economic viability, short-duration crops must be included in the maize–fallow system.

**Methods:**

A field study was conducted in sandy clay loam soil with a randomized complete block design with three replications for three continuous years (2018–2021) under organic management with two crop management practices, *viz.*, (i) conservation agriculture and (ii) conventional agriculture, and six crop diversification options, *viz.*, (i) maize–sweet corn (*Zea mays saccharata*)–vegetable pea (*Pisum sativa*) (M-SC-VP), (ii) maize–sweet corn-mustard (*Brassica juncea*) (M-SC-M), (iii) maize–sweet corn–lentil (*Lens culinaris*) (M-SC-L), (iv) maize–sweet corn–vegetable broad bean (*Vicia faba*) (M-SC-VB), (v) maize (local)–vegetable pea (M-VP), and (vi) maize (local)–fallow (M-F).

**Results:**

The results showed that, the average system productivity was 5.3% lower for conventional agriculture than conservation agriculture. System carbohydrate, protein, fat, dietary fiber, and dietary energy were ~6.9, 6.8, 7.8, 6.7, and 7%, higher in conservation agriculture than in conventional agriculture, respectively. Similarly, system macronutrients (Ca, Mg, P, and K) and system micronutrients yield (Fe, Mn, Zn, and Cu) were, 5.2–8% and 6.9–7.4% higher in conservation agriculture than in conventional agriculture, respectively. On average, over the years, crop diversification with M-SC-VP/M-SC-VB intensive crop rotation had higher system productivity (158%), production efficiency (157%), net returns (benefit–cost ratio) (44%), and dietary net energy returns (16.6%) than the local maize–vegetable pea system. Similarly, the M-SC-VP/M-SC-VB system improved the nutritional security by improving Ca, Mg, P, K, Fe, Mn, Zn, and Cu yield by 35.5–135.7% than the local M-VP system.

**Discussion:**

Conservation agriculture with M-SC-VP/M-SC-VB rotation showed significantly (*p* < 0.05) higher productivity, carbohydrate yield, protein yield, fat yield, and dietary fiber production. It is concluded that conservation agriculture improved soil health and performed better than conventional agriculture in maize-based intensive cropping systems. Overall results indicate that crop diversification with M-SC-VP/M-SC-VB can potentially increase calorie and protein consumption and farm profitability.

## 1. Introduction

Increasing environmental crises and resource degradation had a deleterious effect on food and nutritional security throughout the world. The most significant barrier to attaining sustainable development goals (SDGs), like environmental sustainability and societal wellbeing, is the detrimental impact of poor agricultural production management on ecosystem integrity (1). As a direct consequence of this, both researchers and policymakers are confronted with significant obstacles in their efforts to simultaneously achieve their goals (SDGs) in the areas of food, nutrition, and socioeconomic development. The North Eastern Himalayan (NEH) Region of India is a habitat of ~50 million people, is suffering from low-agriculture production as a result of massive soil degradation and inadequate agronomic management (2–4). In this region, large-scale adoption of mono-cropping generally results in yield stagnation (5), low farm income, and poor resource utilization (6, 7). Maize (*Zea mays* L.)-based cropping system is the second most significant food crop after rice, contributing greatly to household food and nutrition security and livelihoods for low-to middle-income rural and urban populations. These practices at farmers’ fields have less productivity due to poor crop and land management. However, as more land for expanding agriculture is not readily available in these regions, it is becoming immensely important to make use of existing fallow land through diversification and intensification with the adjustment of more crops in cropping sequence. There is an opportunity to improve cropping systems and implement triple cropping rotations in a year, notably by incorporating short-duration sweet corn in the double cropping of maize–legumes/oilseeds. Although most research on cropping systems and conservation agriculture practices in the NEH region focuses on productivity enhancements, nutritional security improvement through efficient intensive sustainable cropping systems for smallholder farming households and rural communities still has scope for research (6, 8).

In the NEH region, rice/maize-based cropping systems are used with extensive tillage (puddling for rice and recurrent tillage in winter crops) and the burning or total removal of crop residues from the field. Maize is grown with moderate to minimal tillage on slopes/terraces. Intensive tillage practices and improper crop management can negatively influence soil quality and potentially cause soil degradation (6, 9, 10). More resource-efficient practices, such as conservation agriculture with full retention of crop residue and diversified crop rotations, are receiving widespread support as potential solutions for lowering the consumption of non-renewable resources, reversing soil deterioration, and restoring soil quality, all while cutting emissions (11–14). Organic intensive cropping system with conservation practices enhanced crop establishment and provision for timely sowing, maintaining, or increasing per unit productivity, lowering production costs, and increasing net returns along with ensuring nutritional security, potential energy availability, and system resilience (15–17), which ensures to achieve little toward the SDGs-3 (good health and wellbeing) and-12 (zero hunger; responsible consumption and production).

The NEH region is best suited for organic farming since the synthetic fertilizers load on the soil is minimal. Agricultural crop residues and organic manure have enormous potential to restore soil health through organic conservation practices (18–20). NEH region produced 2.55 million tonnes of agricultural biomass and has 2.98 million bovines, which stimulates organic crop production. Over the last several decades, there has been a growing emphasis on using conservation agriculture approaches in organic production systems to reduce soil erosion, enhance soil quality, preserve, boost crop yield and nutritional security, and maintain environmental quality (21–24).

In addition, the government of India is placing a strong emphasis on the promotion of organic and natural farming practices. As part of this initiative, national programs such as the Paramparaghat Krishi Vikas Yojana (PKVY; Traditional Agricultural Development Plan), the Rashtriya Krishi Vikas Yojana (RKVY; National Agricultural Development Plan), and the Mission Organic Value Chain Development for North Eastern Regions (MOVCD-NER) are currently being carried out in these regions. In 2023, the Government of India launched the program, i.e., PM PRANAM Yojana (Prime Minister Program for Restoration, Awareness, Nourishment, and Amelioration of Mother earth) to incentivize alternative fertilizers for the promotion of organic farming. Furthermore, the Government of India aims to set up 500 “waste to wealth plants” under GOBAR-DHAN Scheme to convert organic waste into valuable organic nutrient inputs. It is the goal of this initiative to increase the amount of land that is farmed organically by making use of the organic resources that are already in existence, such as manures from livestock, cropping system diversification that includes green manuring, crop residue utilization for soil health restoration, maintaining crop-livestock interactions and crop productivity, and lowering the levels of pollution in the water and air. Because of this, the findings of the current study on the impact of the organic conservation agriculture approach will make it possible for policymakers in the NEH region to put into practice agricultural methods that are efficient.

Few studies have been conducted to analyze the effects of utilizing various types of tillage practices while using double cropping systems, comparing the various conservation agriculture techniques (6, 8). However, robust studies especially on potential nutrition and energy availability, dietary energy returns, or profitability comparing conservation agriculture to conventional agriculture under intensive organic crop diversification systems across the region are lacking. These comparisons would be useful for determining whether conservation agriculture or conventional agriculture is more profitable. A better knowledge of the impacts that conservation agriculture has on crop production, profitability, and nutritional security will assist and explain the performance of these systems and identify the ones that are the most productive and efficient in the region. In light of this, the purpose of this study was to evaluate the organic intensive crop diversification that is the most resilient and sustainable in order to assure the maximum levels of production, profitability, and nutritional security while using the organic conservation agriculture approach in comparison to conventional agriculture.

## 2. Materials and methods

### 2.1. Description of the site and soil characteristics

The location of the experimental site was in the NEH region of the Indian state of Manipur. Most of these regions’ soils are composed of sedimentary rocks, with parent materials originating from the Disang (Eocene) and Barail (Oligocene) groups of sandstone and shale. Intermontane valleys have 2.23 million hectares (~12% of Manipur’s entire geographical area) of cultivable land. During the entire rainy season (April to October), rainfed cereals are mainly grown in the hill and foothill ecosystem (primarily rice and maize) as mono-cropping, with only minor periodic replenishment of plant nutrients from external organic or inorganic sources (5).

The experiment was carried out for three continuous years (2018–2021) at Lapmhel Research Farm (24^ο^49’ N latitude, 93^ο^55’ E longitude, and 786 m above MSL altitude) of the ICAR NEH Region, Manipur Centre, Imphal, India. The research site’s climate is subtropical humid, with a mean (3 years) minimum and maximum temperature variations of 6.2–22.2 and 22.2–30.0°C, respectively. The minimum and maximum relative humidity average varied from 40.0 to 69.9% and 84.6 to 90.9% during experimentation (Supplementary Figure S1). The experimental period of 2018, 2019, and 2020 had a total yearly rainfall of 1326.3, 1147.0, and 1328.9 mm, respectively. The soil at the location of the experiment had the consistency of sandy clay loam. Supplementary Table S1 provides information on the various soil properties that were present at the beginning of the experimentation (0–0.15 m).

### 2.2. Treatment detail and agronomic crop management

The field experiment was conducted in a randomized completely block design (RCBD) with three replications for three continuous years (2018–2021) under organic management with two crop management practices, *viz.*, (i) conservation agriculture and (ii) conventional agriculture, and six crop diversification, *viz.*, (i) maize (*Zea mays*)–sweet corn (*Zea mays saccharata*)–vegetable pea (*Pisum sativa*) (M-SC-VP), (ii) maize–sweet corn–mustard (*Brassica juncea*) (M-SC-M), (iii) maize–sweet corn–lentil (*Lens culinaris*) (M-SC-L), (iv) maize–sweet corn–vegetable broad bean (*Vicia faba*) (M-SC-VB), (v) maize (local)–vegetable pea (M-VP), and (vi) maize (local)–fallow (M-F). In experimental plots of conventional agriculture, four tilling operations were carried out, i.e., two passes of tillage with harrow and two passes of cultivator followed by planking. The crop residues were completely removed from the conventional agriculture plot. In conservation agriculture, reduced tillage operation was maintained with crop residues. In this plot, only one tilling operation was carried out followed by planking with crop residue retention. Except for sweet corn, the above-ground crop leftovers and retained in the field after the economic parts of the crops were collected. Sweet corn biomass is used for livestock feed. The details of the package and practices are given in Table 1.

**Table 1:** Crop-wise package of practices under organic management.

S. No.	Particulars	Maize	Sweet corn	Vegetable pea	Mustard	Lentil	Broadbean	Maize (local)
1.	Variety	HQPM-1	Hi Brix-39	Arkel/Makhyatmubi (local)	M-27	HUL-57	Local Hawaimubi	Local Chaochujak
2.	Seed rate	20 kg ha^−1^	8 kg ha^−1^	80 kg ha^−1^	5 kg ha^−1^	40 kg ha^−1^	100 kg ha^−1^	20 kg ha^−1^
3.	Spacing	60 × 30 cm	60 × 30 cm	40 × 20 cm	40 × 15 cm	30 × 10 cm	30 × 15 cm	60 × 30 cm
4.	Nutrient management	70 kg N from FYM @ 22.58 Mg ha^−1^, 10 kg N from vermicompost @ 1.69 Mg ha^−1^ and 20 kg N from biofertilizers/neem cake (Azotobacter @ 10 kg ha^−1^ + Phosphate solubilizing bacteria @ 10 kg ha^−1^ + Trichoderma @ 5 kg ha^−1^ + Neem cake @ 200 kg ha^−1^)	70 kg N from FYM @ 22.58 Mg ha^−1^, 10 kg N from vermicompost @ 1.69 Mg ha^−1^ and 20 kg N from biofertilizers/neem cake (Azotobacter @ 10 kg ha^−1^ + Phosphate solubilizing bacteria @ 10 kg ha^−1^ + Trichoderma @ 5 kg ha^−1^ + Neem cake @ 200 kg ha^−1^)	14 kg N from FYM @ 4.52 Mg ha^−1^, 2 kg N from vermicompost @ 0.34 Mg ha^−1^ and remaining 4 kg N from biofertilizers/neem cake (Azotobacter @ 10 kg ha^−1^ + Phosphate solubilizing bacteria @ 10 kg ha^−1^ + Trichoderma @ 5 kg ha^−1^ + Neem cake @ 200 kg ha^−1^)	28 kg N from FYM @ 9.03 Mg ha^−1^, 4 kg N from vermicompost @ 0.68 Mg ha^−1^ and remaining 8 kg N from biofertilizers/neem cake (Azotobacter @ 10 kg ha^−1^ + Phosphate solubilizing bacteria @ 10 kg ha^−1^ + Trichoderma @ 5 kg ha^−1^ + Neem cake @ 200 kg ha^−1^)	14 kg N from FYM @ 4.52 Mg ha^−1^, 2 kg N from vermicompost @ 0.34 Mg ha^−1^ and remaining 4 kg N from biofertilizers/neem cake (Azotobacter @ 10 kg ha^−1^ + Phosphate solubilizing bacteria @ 10 kg ha^−1^ + Trichoderma @ 5 kg ha^−1^ + Neem cake @ 200 kg ha^−1^)	14 kg N from FYM @ 4.52 Mg ha^−1^, 2 kg N from vermicompost @ 0.34 Mg ha^−1^ and remaining 4 kg N from biofertilizers/neem cake (Azotobacter @ 10 kg ha^−1^ + Phosphate solubilizing bacteria @ 10 kg ha^−1^ + Trichoderma @ 5 kg ha^−1^ + Neem cake @ 200 kg ha^−1^)	70 kg N from FYM @ 22.58 Mg ha^−1^, 10 kg N from vermicompost @ 1.69 Mg ha^−1^ and 20 kg N from biofertilizers/neem cake (Azotobacter @ 10 kg ha^−1^ + Phosphate solubilizing bacteria @ 10 kg ha^−1^ + Trichoderma @ 5 kg ha^−1^ + Neem cake @ 200 kg ha^−1^)
5.	Pest management	Seed treatment and soil application of Trichoderma harzianum @ 5 kg/ha, Pheromone traps @ 20 traps/ha, Prophylactic application of organic formulations such as Neem oil/Nimbicidine @ 1 ml litre^−1^	Seed treatment and soil application of Trichoderma harzianum @ 5 kg/ha, Pheromone traps @ 20 traps/ha, Prophylactic application of organic formulations such as Neem oil/Nimbicidine @ 1 ml litre^−1^	Seed treatment and soil application of Trichoderma harzianum @ 5 kg/ha, Pheromone traps @ 20 traps/ha, Prophylactic application of organic formulations such as Neem oil/Nimbicidine @ 1 ml litre^−1^	Seed treatment and soil application of Trichoderma harzianum @ 5 kg/ha, Pheromone traps @ 20 traps/ha, Prophylactic application of organic formulations such as Neem oil/Nimbicidine @ 1 ml litre^−1^	Seed treatment and soil application of Trichoderma harzianum @ 5 kg/ha, Pheromone traps @ 20 traps/ha, Prophylactic application of organic formulations such as Neem oil/Nimbicidine @ 1 ml litre^−1^	Seed treatment and soil application of Trichoderma harzianum @ 5 kg/ha, Pheromone traps @ 20 traps/ha, Prophylactic application of organic formulations such as Neem oil/Nimbicidine @ 1 ml litre^−1^	Seed treatment and soil application of Trichoderma harzianum @ 5 kg/ha, Pheromone traps @ 20 traps/ha, Prophylactic application of organic formulations such as Neem oil/Nimbicidine @ 1 ml litre^−1^
6.	Weed management	Two manual weeding at 25 and 50 days after sowing	Two manual weeding at 25 and 50 days after sowing	Two manual weeding at 25 and 50 days after sowing	Two manual weeding at 25 and 50 days after sowing	Two manual weeding at 25 and 50 days after sowing	Two manual weeding at 25 and 50 days after sowing	Two manual weeding at 25 and 50 days after sowing
7.	Water management	--	--	Two live saving irrigation at pre flowing and pod development stage	Two live saving irrigation at pre flowing and pod development stage	Two live saving irrigation at pre flowing and pod development stage	Two live saving irrigation at pre flowing and pod development stage	--

### 2.3. Computation of system productivity

System productivity and maize equivalent production efficiency in terms of maize equivalent yield was computed using Eqs. (1, 2)


(1) $$\mathrm{MEY}\left(\mathrm{Mg/ha}\right)=X+\frac{\left(y^{*}P2\right)}{P1}$$

where *X*: maize grain yield (Mg ha^−1^), *Y*: grain/cob/pod yield of other crops, *viz.*, sweet corn, vegetable pea, mustard, lentil, and broad bean (Mg ha^−1^), P1: selling price of other crops (INR Mg^−1^), and P2: selling price of maize (INR Mg^−1^).

Maize equivalent production efficiency (MEPE) was computed by the following formula:


(2) $$\begin{array}{l} \mathrm{MEPE}=\mathrm{MEY}\left(\mathrm{Mg}{\mathrm{ha}}^{-1}\right)/\\ \mathrm{Duration}\;\mathrm{cropping}\;\mathrm{system}\left(\mathrm{days}\right) \end{array}$$

### 2.4. Computation of carbohydrate, protein, fat, dietary fiber, and nutrients yield

Values of carbohydrate yield, protein yield, fat yield, dietary fiber yield, and nutrient yield are provided in Table 2. The corresponding values were multiplied by the grain yield of respective crops and the system yield was obtained after summation in respective cropping systems. System energy production was calculated from carbohydrate, protein, and fat by multiplying by 4, 4, and 9, respectively (31).

**Table 2:** A total of 100 g of seeds contain nutrients from various crops.

Particulars	Maize	Sweet corn	Vegetable pea	Lentil	Mustard	Broadbean
Moisture%	10.4	76.0	78.9	8.3	8.0	81.0
Carbohydrate (%)	74.3	18.7	14.4	63.4	28.1	11.7
Protein (%)	9.42	3.27	5.42	24.60	26.08	5.60
Fat (%)	4.74	1.35	0.40	1.06	36.24	0.60
Dietary Fiber (%)	7.30	2.00	5.60	10.70	12.20	4.20
Ca/mg	7.0	2.0	25.0	35.0	266.0	22.0
Mg, mg	127.0	37.0	33.0	47.0	370.0	38.0
P, mg	210.0	89.0	108.0	281.0	828.0	95.0
K, mg	287.0	270.0	244.0	677.0	738.0	250.0
Fe, mg	2.71	0.52	1.47	6.51	9.21	1.90
Mn, mg	0.49	0.16	0.41	1.39	2.45	0.32
Zn, mg	2.21	0.46	1.24	3.27	6.08	0.58
Cu, mg	0.31	0.05	0.18	0.75	0.65	0.07
Energy, calories	377.5	100.0	82.9	361.5	542.8	74.6
Reference	(25)	(26)	(27)	(28)	(29)	(30)

### 2.5. Potential energy availability

Potential energy availability (PEA) is computed by the following formula:


(3) $$\begin{array}{l} \mathrm{PEA}\;\;\left(\mathrm{Person/ha/year}\right)\\ =\mathrm{System}\;\;\mathrm{energy}\;\;\mathrm{yield}\;\;\left(\mathrm{Kcal}\right)/\\ \mathrm{average}\;\;\mathrm{requirement}\;\;\mathrm{of}\;\;\mathrm{energy}\;\;\mathrm{per}\;\;\mathrm{person} \end{array}$$

where the energy requirement for men is 2,710 kCal, for women is 2,130 kCal, and the average is 2,420 kCal, which is considered for the PEA calculation (32).

### 2.6. Computation of economics

For economic analysis, the total cost of production, the total return from the system’s outputs (main and by-products), gross return, benefit-to-cost ratio, and dietary energy returns were computed using Eqs. (4–7), where the total variable cost of production was considered as the total cost of production. The minimum support price (MSP) was considered as per the prevailing market price in INR (Indian rupees) for maize, sweet corn, vegetable pea, mustard, lentil, and broad bean. The details of prices for accounting of economics are presented in Supplementary Table S2.


(4) $$\begin{array}{l} \mathrm{Gross}\;\;\mathrm{retrun}\;\;\left(\mathrm{GR,}\;\;\mathrm{INR}\;\;{\mathrm{ha}}^{-1}\right)\\ =\mathrm{System}\;\;\mathrm{output}\;\;\left(\mathrm{Mg}\;\;{\mathrm{ha}}^{-1}\right)\\ \times \mathrm{output}\;\;\mathrm{price}\;\;\left(\mathrm{INR}\;\;{\mathrm{Mg}}^{-1}\right) \end{array}$$

(5) $$\begin{array}{l} \mathrm{Net}\;\;\mathrm{return}\;\;\left(\mathrm{NR,}\;\;\mathrm{INR}\;\;{\mathrm{ha}}^{-1}\right)\\ =\mathrm{System}\;\;\mathrm{output}\;\;\left(\mathrm{Mg}\;\;{\mathrm{ha}}^{-1}\right)-\\ \mathrm{Total}\;\;\mathrm{cost}\;\;\mathrm{of}\;\;\mathrm{production}\;\;\left(\mathrm{INR}\;\;{\mathrm{ha}}^{-1}\right) \end{array}$$

(6) $$\begin{array}{l} \mathrm{Benefit}\;\;\mathrm{to}\;\;\mathrm{cost}\;\;\mathrm{ratio}\;\;\left(\mathrm{BCR}\right)\\ =\mathrm{Total}\;\;\mathrm{return}\;\;\left(\mathrm{INR}\;\;{\mathrm{ha}}^{-1}\right)\\ /\mathrm{Total}\;\;\mathrm{cost}\;\;\mathrm{of}\;\;\mathrm{production}\;\;\left(\mathrm{INR}\;\;{\mathrm{ha}}^{-1}\right) \end{array}$$

(7) $$\begin{array}{l} \mathrm{Dietary}\;\;\mathrm{energy}\;\;\mathrm{returns}\;\;\left(\mathrm{Kj}\;\;{\mathrm{INR}}^{1}\mathrm{investment}\right)\\ =\mathrm{Energy}\;\;\mathrm{yield}\;\;\left(\mathrm{Kj}\;\;{\mathrm{ha}}^{-1}\right)\\ /\mathrm{Total cost of production (INR/ha)} \end{array}$$

### 2.7. Data analysis

The data from grain yield and carbohydrate yield, protein yield, fat yield, dietary fiber yield, and nutrients yield were processed for analysis of variance (ANOVA) in a factorial RCBD using R version 9.2 to examine the statistical significance of the treatments (crop management and crop diversification). Using SPSS version 16.0, the LSD of the mean was calculated using Duncan’s multiple range test (DMRT) (*p* < 0.05).

## 3. Results

### 3.1. System productivity and production efficiency

Averaged over the 3 years (2018–2021), conservation agriculture significantly (*p* < 0.05) produced 5.3% higher system productivity (15.8 Mg ha^−1^) than conventional agriculture (15.0 Mg ha^−1^). Among the crop diversification options higher system productivity was recorded in M-SC-VB (22.8 Mg ha^−1^) ≥ M-SC-VP (22.6 Mg ha^−1^) than the popularized cropping system on farmers’ fields as maize–fallow (M-F: 3.2 Mg ha^−1^) and maize–vegetable pea (local) (M-VP; 8.8 Mg ha^−1^) (Table 3). Conservation agriculture enhanced production efficiency by 5.1% than conventional agriculture. On average, the highest production efficiency was recorded in M-SC-VB (62.5 kg ha^−1^ day^−1^) followed by M-SC-VP (61.9 kg ha^−1^ day^−1^) than the popularized cropping system of M-VP (8.7 kg ha^−1^ day^−1^) and M-F (3.7 kg ha^−1^ day^−1^; Table 3).

**Table 3:** Effect of crop management and cropping system on system productivity, production efficiency, and economics in the maize-based cropping system.

Treatments	System productivity (Mg ha^−1^)	Production efficiency (Kg ha^−1^ day^−1^)	Net returns (INR x 10^3^ ha^−1^)	Benefit–cost ratio	Dietary energy returns, Kj INR^−1^ invested
Agronomic management					
Conservation agriculture	15.8	43.2	242.7	2.49	280.3
Conventional agriculture	15.0	41.1	227.4	2.23	247.5
LSD (*p* < 0.05)	0.72	1.91	14.3	0.12	11.48
Crop diversification					
M-SC-VP	22.6	61.9	353.5	2.99	276.4
M-SC-M	16.9	46.3	255.2	2.35	268.9
M-SC-L	17.9	49.1	273.8	2.52	276.1
M-SC-VB	22.8	62.5	356.2	2.97	259.2
M-VP	8.8	24.2	130.7	2.07	229.6
M-F	3.2	8.7	41.2	1.25	273.3
LSD (*p* < 0.05)	1.4	3.7	33.8	0.20	20.7

M-SC-VP, maize–sweet corn–vegetable pea; M-SC-M, maize–sweet corn–mustard; M-SC-L, maize–sweet corn–lentil; M-SC-VB, maize–sweet corn–vegetable broad bean; M-VP, maize (local)–vegetable pea (local); M-F, maize (local)–fallow, LSD, least significant difference.

### 3.2. Dietary carbohydrate, protein, fat, and fiber yield

**Figure 1:**
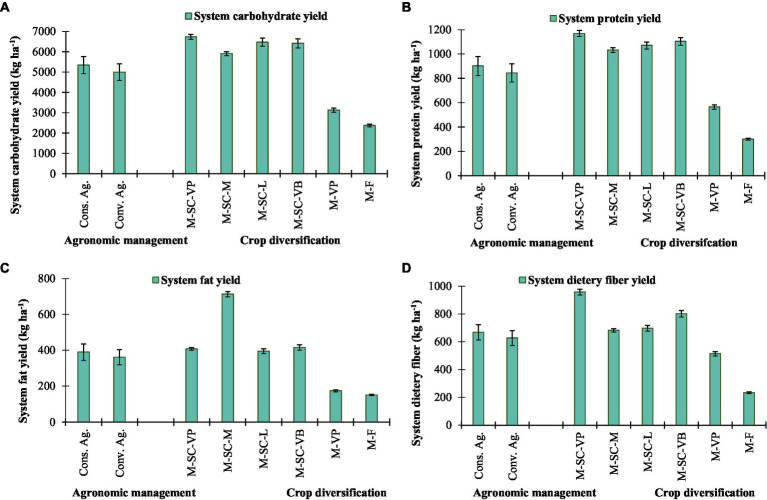
Effect of crop management and cropping system on **(A)** system carbohydrate yield, **(B)** system protein yield, **(C)** system fat yield, and **(D)** system dietary fiber yield. The vertical bar represents the standard error of the mean (*p* < 0.05). Cons. Ag., Conservation agriculture; Conv. Ag., conventional agriculture; M-SC-VP, maize–sweet corn–vegetable pea; M-SC-M, maize–sweet corn–mustard; M-SC-L, maize–sweet corn–lentil; M-SC-VB, maize–sweet corn–vegetable broadbean; M-VP, maize–vegetable pea; M-F, maize–fallow.

Averaged over the years, conservation agriculture significantly recorded 6.8, 6.9, 7.8, and 6.7% higher dietary carbohydrate, protein, fat, and fiber yield than conventional agriculture (Figure 1). Among the cropping system, the highest dietary carbohydrate (6727.4 kg ha^−1^), dietary protein (1168.5 kg ha^−1^), and dietary fiber (956.5 kg ha^−1^) are observed in M-SC-VP followed by M-SC-VB cropping system, while highest dietary fat was obtained in M-SC-M cropping system and it was comparable with M-SC-VB and M-SC-VP (Figure 1). The minimum dietary carbohydrate, protein, fat and fiber yield were recorded in M-F and M-VP than other cropping systems, where additional sweet corn adjusted in system.

### 3.3. Dietary essential mineral yield

**Figure 2:**
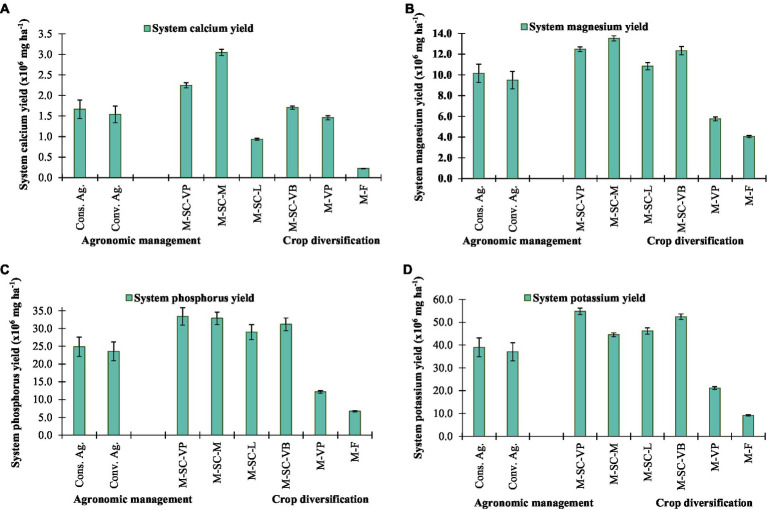
Effect of crop management and cropping system on mineral yield of **(A)** calcium, **(B)** magnesium, **(C)** phosphorus, and **(D)** potassium. The vertical bar represents the standard error of the mean (*p* < 0.05). Cons. Ag., Conservation agriculture; Conv. Ag., conventional agriculture; M-SC-VP, maize–sweet corn–vegetable pea; M-SC-M, maize–sweet corn–mustard; M-SC-L, maize–sweet corn–lentil; M-SC-VB, maize–sweet corn–vegetable broadbean; M-VP, maize–vegetable pea; M-F, maize–fallow.

**Figure 3:**
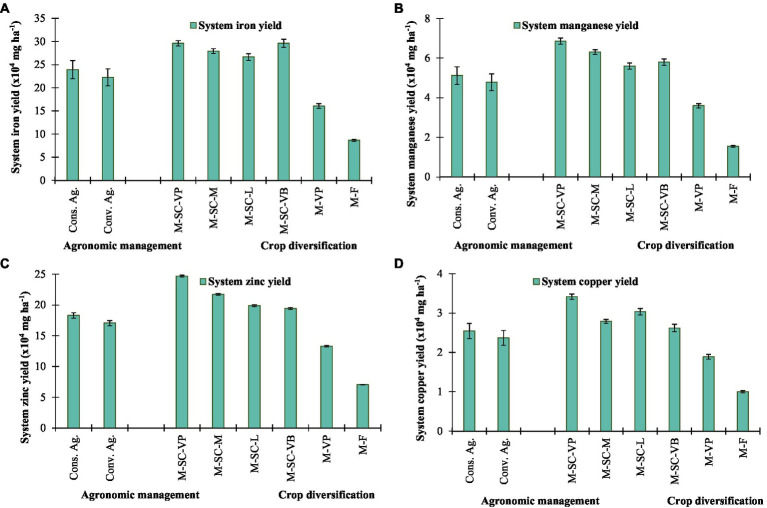
Effect of crop management and cropping system on trace mineral yield of **(A)** iron, **(B)** manganese, **(C)** zinc, and **(D)** copper. The vertical bar represents the standard error of the mean (*p* < 0.05). Cons. Ag., Conservation agriculture; Conv. Ag., conventional agriculture; M-SC-VP, maize–sweet corn–vegetable pea; M-SC-M, maize–sweet corn–mustard; M-SC-L, maize–sweet corn–lentil; M-SC-VB, maize–sweet corn–vegetable broadbean; M-VP, maize–vegetable pea; M-F, maize–fallow.

Cropping system diversification with sustainable agronomic management practices significantly influenced the nutritional yield of Calcium (Ca), magnesium (Mg), phosphorus (P), potassium (K), iron (Fe), manganese (Mn), zinc (Zn), and copper (Cu). Conservation agriculture enhanced the essential minerals yield of Ca, Mg, P, K, Fe, Mn, Zn, and Cu by 8.0, 7.0, 5.6, 5.2, 7.4, 6.9, 7.2, and 7.4% than conventional agriculture (Figures 2, 3). Among cropping systems, the highest essential nutrients harvest, *viz.*, P (33.4 × 10^6^ mg ha^−1^), K (54.8 × 10^6^ mg ha^−1^), Fe (29.6 × 10^4^ mg ha^−1^), Mn (6.9 × 10^4^ mg ha^−1^), Zinc (24.7 × 10^4^ mg ha^−1^), and Cu (3.42 × 10^4^ mg ha^−1^) were obtained in M-SC-VP cropping rotation, except for Ca (3.05 × 10^6^ mg ha^−1^) and Mg (13.5 × 10^6^ mg ha^−1^), which were obtained in M-SC-M cropping rotation. The minimum essential nutrient production was obtained in M-F and M-VP cropping rotations (Figures 2, 3).

### 3.4. Energy production and potential energy availability

**Figure 4:**
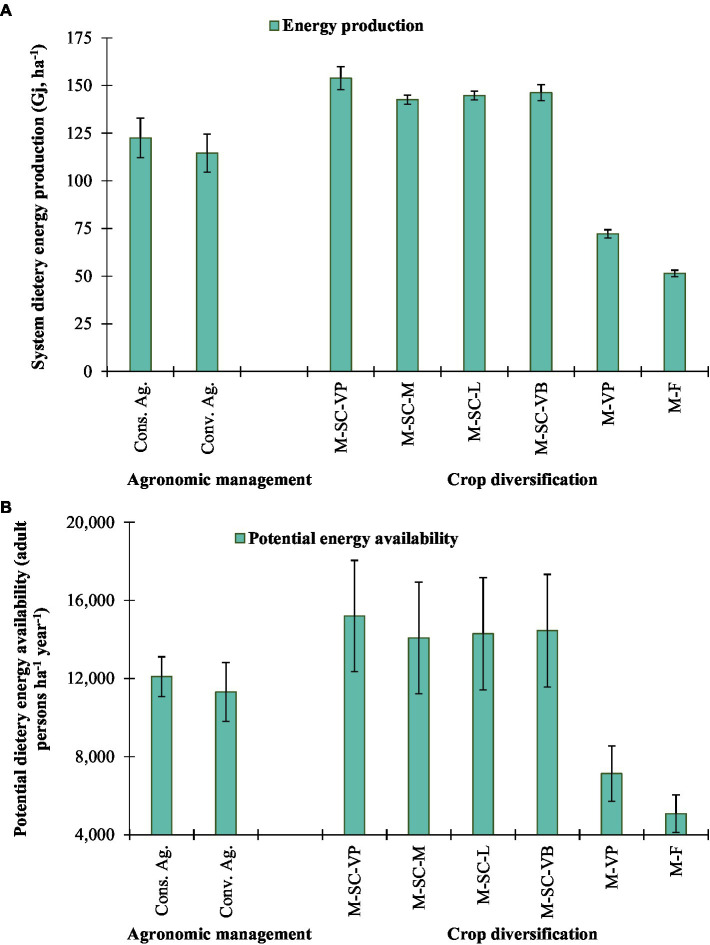
Effect of crop management and cropping system on **(A)** system dietary energy production and **(B)** potential dietary energy availability. The vertical bar represents the standard error of the mean (*p* < 0.05). Cons. Ag., Conservation agriculture; Conv. Ag., conventional agriculture; M-SC-VP, maize–sweet corn–vegetable pea; M-SC-M, maize–sweet corn–mustard; M-SC-L, maize–sweet corn–lentil; M-SC-VB, maize–sweet corn–vegetable broadbean; M-VP, maize–vegetable pea; M-F, maize–fallow.

Conservation agriculture practices (122.5 Gj ha^−1^) recorded the highest dietary energy production than conventional agriculture (114.5 Gj ha^−1^) (Figure 4). Consequently, in the same treatment, the higher potential energy availability was recorded for 12,094 persons ha^−1^ year^−1^ than conventional agriculture (11,310 persons ha^−1^ year^−1^). Among the cropping diversification, the highest dietary energy production was recorded in M-SC-VP (153.9 Gj ha^−1^) followed by M-SC-VB (146.3 Gj ha^−1^; Figure 4). M-SC-VP cropping rotation could fulfill the dietary energy requirement in terms of PEA of 15,199 persons ha^−1^ year^−1^ followed by M-SC-VB (14,444 persons ha^−1^ year^−1^). The minimum energy production was obtained in M-F (51.4 Gj ha^−1^) followed by M-VP (72.2 Gj ha^−1^). Similarly, M-F and M-VP could fulfill the dietary energy requirement for only 5,073 and 7,128 persons ha^−1^ year^−1^, respectively (Figure 4).

### 3.5. Production economics

Agronomic management practices significantly influence the farm net returns of maize-based cropping diversification. The highest net returns and B:C ratio were recorded in Indian rupees (INR) 242.3 × 10^3^ and 2.49 in conservation agriculture than conventional agriculture INR 227.4 × 10^3^ ha^−1^ and 2.23, respectively. The cropping system significantly (*p* < 0.05) differed the net returns and B:C ratio. The M-S-VB gave the highest net returns of INR 356.2 × 10^3^ ha^−1^ followed by M-SC-VP (INR 353.5 × 10^3^ ha^−1^). However, the M-SC-VB system enhanced the net farm income by 764 and 172% over M-F and M-VP cropping sequences, respectively. Similarly, M-SC-VP performed equally better and recorded significantly higher farm net income by 758 and 170% than M-F and M-VP, respectively. Similarly, the higher benefit–cost ratio was recorded in M-SC-VP (2.99) ≥ (2.97) than the dominant cropping system of M-VP (2.07) and M-*F* (1.25) (Table 3).

### 3.6. Dietary energy returns

Dietary energy returns in terms of energy produced per INR invested significantly (*p* < 0.05) influenced due to agronomic management and cropping diversification. Averaged over the 3 years, conservation agriculture improved 13.25% dietary energy returns than conventional agriculture. Among cropping diversification, the highest dietary energy returns were obtained in M-SC-VP (280.3 Kj INR^−1^ invested). The least dietary energy return was obtained in M-VP (229.6 Kj INR^−1^ invested; Table 3).

## 4. Discussion

### 4.1. Current and future importance of organic conservation agriculture in the north eastern Himalayas

There are seven states that constitute the NEH region of India, these states are Assam, Arunachal Pradesh, Manipur, Mizoram, Nagaland, Meghalaya, and Sikkim. The NEH has a total geographical area of ~18.37 million hectares (M ha) (33), and its net cultivated area is 1.77 M ha (34). The NEH is the most ideal niche location for the development of organic crop production. Because of this, fertilizer usage in the NEH states is almost negligible, with the exception of Manipur (68.3 kg ha^−1^), in contrast to the overall fertilizer use in India, which is 133 kg ha^−1^ (35). As a result, agricultural crop residues have a tremendous amount of potential to enhance the quality of the soil in the NEH region. Crop residues are a by-product of crop production in NEH of India (2.55 million tonnes of crop residues include 0.40 M t in Arunachal Pradesh, 0.90 M t in Manipur, 0.51 M t in Meghalaya, 0.06 M t in Mizoram, 0.49 M t in Nagaland, 0.04 M t in Tripura, and 0.15 M t in Sikkim). The residues of rice and maize are not being utilized as livestock feed, rather 11% (0.28 M t) of total crop residues are burnt causing pollution, especially air (36). These residues may be used for crop production under organic conservation agriculture.

The total bovine population in NEH is estimated to be ~2.98 million, with bovine (mostly cattle and buffalo) providing most of the manure used in agricultural production. To cash these opportunities to convert animal waste into wealth, the Government of India launched the Galvanizing Organic Bio-Agro Resources (GOBAR)-DHAN scheme under Swachh Bharat Mission (Gramin). To encourage organic farming, Indian Government launched the PM PRANAM Yojana to incentivize the alternative options of chemical fertilizers. The traditional symbiotic interactions between crops and livestock in smallholder, mixed farming systems have been disrupted as a result of several factors. These factors include the gradual transition away from the use of draft animals in favor of electrical and mechanical sources of power, the decreasing reliance on crop residues as ruminant fodder, the large-scale burning of straw, and the gradual decline in recycling farmyard manure to enrich soils. There are still some locations in the NEH that use crop residues as feed for animals, although these areas are becoming increasingly rare. According to the findings of the latest research, this indicates that there is a significant possibility to make use of the crop residues that are present in the NEH region in order to promote organic conservation agriculture practices. In this way, the soil’s potential for long-term productivity could be preserved.

Over the course of the last few decades, there has been a growing awareness of the importance of utilizing conservation agriculture systems in order to reduce the amount of soil erosion, enhance the quality of the soil, maintain, or increase crop productivity and nutritional security, and keep the environmental quality intact in agricultural systems (6). In order to improve carbon sequestration in agricultural land and reduce green house gas (GHG) emissions, one of the most important factors is the quantity and quality of crop residues that are provided *via* cultivation using conservation agriculture (no-tillage) (37). It is commonly believed that the conservation tillage system can improve soil quality by increasing soil health indicators and, therefore, the functioning of the soil microbial community, which is important for the transformation and mineralization of organic compounds and nutrients in soil ecosystems. Conservation agriculture system involves minimal physical disturbance and soil inversion (38). In the NEH region, the area is suffering from a catastrophic loss of plant cover and top fertile soils as a direct result of the extreme erosion caused by steep slopes.

Practicing shifting cultivation in a 0.756 million ha land area resulted in burning phytomass (including forest floors) of more than 8.5 million tonnes annually (39). This has resulted from disturbances in soil carbon dynamics, mostly due to the loss of topsoil from surface runoff in sloping lands. In this context, conservation agriculture offers a significant, multi-dimensional opportunity to transform large-scale agricultural waste streams from financial and environmental liability to valuable assets. If the agriculture crop biomass is utilized through conservation agriculture, millions of tonnes of carbon can be sequestered and fertile soil will be saved from erosion. In shifting cultivation, instead of “slash and burn,” the practice should be “slash and recycle biomass.” In addition, the government of India is placing a strong emphasis on the promotion of organic and natural farming practices. As a part of this initiative, national programs such as the Paramparaghat Krishi Vikas Yojana (PKVY; Traditional Agricultural Development Plan), the Rashtriya Krishi Vikas Yojana (RKVY; National Agricultural Development Plan), and the Mission Organic Value Chain Development for North Eastern Regions(MOVCD-NER) are currently being carried out. The goal of this initiative to increase the amount of land that is farmed organically by making use of the organic resources that are already in existence, such as manures from livestock, cropping system diversification that includes green manuring, crop residue utilization for soil health restoration, maintaining crop–livestock interactions and crop productivity, and lowering the levels of pollution in the water and air. Because of this, the findings of the current study on the impact of organic conservation measures will make it possible for policymakers in the NEH area to put into practice agricultural methods that are efficient.

### 4.2. Production efficiency

Agricultural management strategies those are sustainable from an ecological perspective are essential to the continued provision of ecosystem services (4, 40). The designing of a food and nutrient-efficient cropping system to sustain the livelihood of farmers will achieve little toward SDGs-3 (good health and wellbeing) and-12 (zero hunger; responsible consumption and production). The utilization of leguminous crops within the system and the recycling of biomass led (17.2–18.4 tonnes ha^−1^; Supplementary Figure S1) to an increase in the production efficiency per unit of land following the implementation of conservation agriculture. The retention of more residues of above-ground biomass helps to improve the quality of the soil’s properties (18, 41). Therefore, production efficiency and overall system productivity increased by ~5% due to the retention of residues as compared to the removal of residues in conventional agriculture (6). The possibility to fulfill SDGs, particularly those related to good health and wellbeing, is offered through higher production efficiency and system productivity. In a similar manner, M-SC-VP and M-SC-VB offered a system productivity and production efficiency that was approximately 2.6 and 7.0 times greater than that of the most often used M-F and M-VP cropping systems, respectively.

### 4.3. Dietary productivity of carbohydrate, protein, fat, fiber, and mineral

The maize–sweet corn–vegetable pea/broad bean intensive cropping system in the NEH region produces a higher dietary carbohydrate (107–115%), dietary protein (96–105%), dietary fat (134–138%), dietary fiber (56–86%), dietary minerals like calcium (17–54%), magnesium (114–116%), phosphorus (156–175%), potassium (148–159%), iron (84%), manganese (61–90%), zinc (46–86%), and copper (39–81%) than the most popular local M-VP cropping rotation. In addition, the designing of intensive cropping diversification improved and ensured greater availability of dietary foods and minerals than locally adopted systems, such as local M-VP and M-F cropping diversification. These intensive cropping rotations also reduced the area of land needed for agricultural production. In accordance with the United Nations Sustainable Development Goals, the Northeast Himalayan region faces the challenge of maximizing sustainable agricultural development while simultaneously increasing grain production to a level that satisfies dietary needs for carbohydrates, protein, fat, fiber, and minerals while simultaneously reducing resource use (8, 42, 43). The calorie and protein yields of all crops grown throughout the Kharif, Rabi, and summer seasons, as well as the yields of all cropping systems, were considerably impacted by conservation agriculture. The residue retention of maize and subsequent season crops resulted in an increase of 5–8% in the average output of dietary carbohydrates, proteins, fats, fibers, and minerals. These findings are consistent with those of earlier research conducted in South Asia, where the implementation of conservation agriculture-based management strategies has led to a 3.0–6.0% increase in protein production across a number of cropping systems (44, 45). The greater calorie and protein yields that were achieved *via* the use of conservation agriculture were direct results of the higher grain yield that was achieved through the use of these management strategies. The adoption of appropriate agricultural patterns in conjunction with the utilization of appropriate technology has the potential to enhance the calorie and protein security of smallholder farmers in India and South Asia (44).

### 4.4. Dietary energy and potential energy availability

The production of food is dependent on ecosystems in which the soil should be in good condition and working properly, which in turn gives services to agriculture by fertilizing the soil with the necessary organic inputs (16, 46). These ecosystem services, which include regulating and providing support, make delivering ecosystem services possible (47). The recycling of agricultural residues and intensification of crop production with a diversity of different crops contribute to an increase in the quality of ecosystem services. The production of food that is required to fulfill nutritionally adequate diets is reliant on the good functioning of the ecosystem, which is based, in turn, on the variety of farming techniques and inputs. India has attained food self-sufficiency or food security in terms of *per capita* calorie availability during the past decade due to its constant and sustainable development in food production. This feat was accomplished over the past 10 years (48). Despite this, the need for sustainable intensification and diversification of sole cropping systems or double to triple cropping systems with manipulation of agronomic management practices under changing climate scenarios and ensuring the food and nutrition security of an increasing population will continue to remain the major challenge in Indian agriculture. This will continue to be the case as long as the population of India continues to rise (49).

Our research has the potential to contribute to the process of formulating policies and establishing strategies for achieving and maintaining food and nutritional security through the sustainable intensification and diversification of crop production. Dietary patterns have been consistently shifting in the NEH region ever since there has been a shift in attitude toward healthy (organic) and complete dietary food. This shift has resulted in a greater emphasis on the consumption of foods that are high in carbohydrates, proteins, dietary fiber, and enriched mineral content. This study investigated the dietary energy and potential energy availability (PEA) for the food intake from the comparative cropping system under conventional agriculture and conservation tillage. The analysis was based on the changes in diet. The results of our research indicated that the utilization of intensive cropping systems (M-SC-VP, M-SC-M, M-SC-L, and M-SC-VB) led to a higher production of dietary energy by 103 and 186%, respectively, when compared to the conventional cropping system, which consisted of growing maize as sole and in conjunction with vegetable peas. Among the systems, M-SC-VP and M-SC-VB had the best overall performance and the highest PEA. This guarantees that the requirements for dietary energy are needed by 15,199–14,444 adult person year^−1^. The maize–sweet-corn–vegetable pea/broad bean system that was implemented with conventional tillage resulted in the maximum yields of grain, calories, carbohydrates, proteins, and minerals at the system level. In the NEH region, maize does not directly contribute to the composition of human meals. On the contrary, this is a significant source of poultry feed, and chicken is one of the important sources of protein in the diets of people who live in that region. Therefore, there is potential to integrate maize into human diets and to increase its consumption by altering the dietary patterns of human consumers. Our research has shown that implementing these systems into conventional farming to make the most efficient use of available resources is one way to enhance the level of food and nutritional security available to the region’s growing population.

### 4.5. Dietary energy returns and economics

It is crucial to know how smallholder farmers in the NEH area may optimize their dietary energy returns and farm profitability through the efficient and effective use of natural resources in the intensive cropping systems that are used in that region (land, water, energy, and labor). This study examined the impact of six different cropping diversifications and two different alternative options (conservation and conventional) on the productivity of the systems, as well as the nutritional supply and profitability of the systems. Because of the high yields of hybrid maize and the adjustment of one short-duration sweet corn crop that was grown in the system, the maize–sweet corn–vegetable pea/broad bean rotation resulted in a higher net margin (~157%) and dietary energy returns (~20%) than the maize–vegetable pea cropping systems. This was due to the fact that the rotation included maize, sweet corn, vegetable peas, and broad beans. It is consistent with the findings of past studies conducted in the NEH region that the intensive cropping system has a larger gross margin than the conventional double and sole cropping systems (6).

## 5. Conclusion

Increasing cereal-based cropping systems’ productivity, profitability, and long-term sustainability is a challenge for NEH’s low-to middle-income rural and urban populace. This study showed that maize–sweet corn–vegetable pea/broad bean systems could increase systems productivity, production efficiency, carbohydrate yield, protein yield, dietary fiber yield, and grain calories by 158, 157, 110, 101, 71.3, and 108% while providing 171.5, 44, and 16.6% higher net margin, benefit–cost ratio, and dietary energy returns per INR invested than the local farmer practice of maize–vegetable pea system. Four cropping systems (excluding the local maize–vegetable pea and maize–fallow system) evaluated here might benefit from conservation tillage. The maize–sweet corn–vegetable pea/broad bean system had higher Ca, Mg, P, K, Fe, Mn, Zn, and Cu nutritional security and PEA than the other system. This study shows that conservation agriculture-based management approaches can benefit maize-based intensive rotations in a subtropical environment of the NEH. Although the study farm is bordered by farmers’ fields with comparable climates and soil, crop management procedures in research stations might differ according to natural factors and socio-economic contexts. Depending on farmers’ priorities and risk tolerance, our findings propose a basket of technology solutions for smallholders to implement conservation agriculture. The development aims in the NEH as a whole include extending these approaches from research the field of smallholder farmers.

## 6. Policy implications

Sustainable development goals aim for promoting responsible production and consumption which also include food without any chemical residues. Government of India aim to bring 10% of the net cultivated area under certified organic farming by 2030 which is currently only 3%. The introduction of farmer-friendly certification systems such as the Participatory Guarantee System (PGS) and large area certification also encourages farmers to adopt alternative production systems. Focus is being made on the promotion of chemical-free farming in niche areas (with low consumption of fertilizers and pesticides; for example, north-eastern states, hilly areas in other parts, etc.) and niche crops (crop responding to organic management). Therefore, findings from the study can be integrated with ongoing government-sponsored promotional schemes like the PKVY, Traditional Agricultural Development Plan, the RKVY, National Agricultural Development Plan, and the MOVCD-NER to reap better benefits. Further alternative fertilizing strategies for organic farming can also be integrated through the PM PRANAM Yojana which will encourage organic growers. Furthermore, the Government of India aims to set up 500 “waste to wealth plants” GOBAR-DHAN scheme to convert organic waste into valuable assets (an organic source of nutrients). Soil and human nutritional security can be achieved through the promotion of science-led organic farming including choosing appropriate tillage options along with cropping systems as evident from the study. Prioritized solutions for organic farming will improve productivity leading to better export of safe food to the global market and benefit the larger population looking for chemical residue-free food.

## Data availability statement

The original contributions presented in the study are included in the article/Supplementary material, further inquiries can be directed to the corresponding authors.

## Author contributions

MA: conceptualization, methodology, investigation, monitoring, data curation, and writing of original and final draft. NR: review and writing, editing, and project administration. MA: writing of original first draft, review and editing, and data analysis. SB: review, writing, and editing. JL: review, writing, and editing. AP: review, editing, and project administration.

## Funding

The authors gratefully acknowledge ICAR-IIMR, Ludhiana and ICAR-IIFSR, Modipuram (AI-NPOF project) for financial assistance under collaborative project. We are thankful to the Director, ICAR-IIFSR, Modipuram for financial assistance in the publication of research outcomes under the AI-NPOF project. We are grateful to Frontiers for APC support.

## Conflict of interest

The authors declare that the research was conducted in the absence of any commercial or financial relationships that could be construed as a potential conflict of interest.

## Publisher’s note

All claims expressed in this article are solely those of the authors and do not necessarily represent those of their affiliated organizations, or those of the publisher, the editors and the reviewers. Any product that may be evaluated in this article, or claim that may be made by its manufacturer, is not guaranteed or endorsed by the publisher.

## Abbreviations

NEH: North Eastern Himalaya
M-SC-VP: Maize–sweet corn–vegetable pea
M-SC-M: Maize–sweet corn–mustard
M-SC-L: Maize–sweet corn–lentil
M-SC-VB: Maize–sweet corn–vegetable broad bean
M-VP: Maize–vegetable pea
M-F: Maize–fallow
PM PRANAM: Prime Minister Program for Restoration, Awareness, Nourishment, and Amelioration of Mother earth
GOBAR-DHAN: Galvanizing Organic Bio-Agro Resources-DHAN
PKVY: Paramparaghat Krishi Vikas Yojana (Traditional Agricultural Development Plan)
RKVY: Rashtriya Krishi Vikas Yojana (National Agricultural Development Plan)
MOVCD-NER: Mission Organic Value Chain Development for North Eastern Regions
SDGs: Sustainable development goals
MEPE: Maize equivalent production efficiency
MEY: Maize equivalent yield
PEA: Potential energy availability
Gj: Giga joule
kcal: Kilo calorie
